# Mechanism of TBK1 activation in cancer cells

**DOI:** 10.1016/j.cellin.2024.100197

**Published:** 2024-08-22

**Authors:** Lianxin Hu, Qing Zhang

**Affiliations:** a Department of Urology and Institute of Urologic Science and Technology, The First Affiliated Hospital, Zhejiang University School of Medicine, Hangzhou, 310003, Zhejiang, China; b Department of Pathology, University of Texas Southwestern Medical Center, Dallas, TX, 75390, USA; c Simmons Comprehensive Cancer Center, University of Texas Southwestern Medical Center, Dallas, TX, 75390, USA

**Keywords:** TBK1, Ser172 phosphorylation, Cancer

## Abstract

TANK-binding kinase 1 (TBK1) is a serine/threonine kinase with well-established roles as a central player in innate immune signaling. Dysregulation of TBK1 activity has been implicated in a variety of pathophysiologic conditions, including cancer. Generally, TBK1 acts as an oncogene and increased TBK1 activity, indicated by increased phosphorylation at the Ser172 residue, can be observed in multiple human cancers. TBK1 can be activated either by autophosphorylation of Ser172 or transphosphorylation at this site by upstream kinases. Serving as a hub for integrating numerous extracellular and intracellular signals, TBK1 can be activated through multiple signaling pathways. However, the direct upstream kinase responsible for TBK1 activation remains elusive, which limits our comprehensive understanding of its activation mechanism and potential therapeutic application targeting TBK1-related signaling especially in cancer. In this review, we summarize the findings on mechanisms of TBK1 activation in cancer cells and recent discoveries that shed light on the direct upstream kinases promoting TBK1 activation.

## 1 Introduction

The serine/threonine protein kinase TBK1, discovered two decades ago as a member of the IκB kinase (IKK) protein kinase family, plays crucial roles in regulating innate immune response by promoting type I interferon (IFN) and modulating NF-κB signaling (Tojima et al., 2000; ZHAO & ZHAO, 2019). Recently additional functions of TBK1 have been uncovered, including regulation of cell proliferation, survival, cell death, signaling transduction and metabolism (Revach et al., 2020; Zhou et al., 2020). Alongside these discoveries, many new TBK1 substrates have been identified. Dysregulation of TBK1 has been linked to various human diseases, including cancer.

In addition to directly regulate cancer cell proliferation and survival, TBK1 plays a significant role in promoting tumorigenesis by altering the function of immune cells within the tumor microenvironment. Multiple large-scale CRISPR screens have identified TBK1 as a candidate immune evasion gene (Lawson et al., 2020; Manguso et al., 2017; Vredevoogd et al., 2020). Targeting TBK1 can effectively overcome immune checkpoint blockade resistance, partially due to preventing cytokines-induced, RIPK1-dependent cell death (Sun et al., 2023). The study of TBK1's function in immune cells within tumor microenvironment has been emerging. TBK1 signaling has been shown to restrain inflammatory responses in macrophages and myeloid cells (Gao et al., 2022). Furthermore, dendritic cell-specific deletion of TBK1 can enhance antitumor immunity in murine models (Xiao et al., 2017). Current data also suggest that TBK1 may influence T cell differentiation and activation. *In vivo* administration of TBK1 inhibitor was shown to promote the accumulation of early effector CD8^+^ T cells, with associated reduction of terminally exhausted effector CD8^+^ T cells—a phenotype similarly observed in patients with hypomorphic TBK1 mutations (Sun et al., 2023; Taft et al., 2021). For a comprehensive review of TBK1's role in tumor immunity, please see (Miranda et al., 2024). Here we will focus on TBK1 activity specifically in cancer cells.

Despite the growing interest of studying TBK1 in cancer biology and immunology, the precise mechanisms governing TBK1 activation in cancer cells remain incompletely understood. A remarkable feature of TBK1 is its substrate specificity in response to different upstream signaling (Helgason et al., 2013). For example, activation of TBK1 by DNA or RNA virus infection can induce IRF3 phosphorylation and translocation into nucleus, while autophagy mediated TBK1 activation can promote phosphorylation of the autophagy receptors like p62 and OPTN. Various adaptor proteins can associate with TBK1 and recruit it to different signaling complexes (Helgason et al., 2013), where it can be activated either by autophosphorylation or by transphosphorylation. The phosphorylation of Ser172 residue on TBK1 is critical and is commonly used as an indicator for its activity. Phosphorylated Ser172 coordinates with Arg162 in the activation loop, Arg134 in the catalytic loop and Arg54 in C-helix of TBK1 to reorganize the activation loop from an inactive conformation, enabling substrate binding and phosphotransfer reaction (Larabi et al., 2013).

However, a key question regarding TBK1 activation is to identify the initial trigger for the phosphorylation of Ser172 before the signal amplification step. This issue has been discussed in detail in a preprint manuscript (Ye et al., 2022). Briefly speaking, TBK1 works in the form of homodimer with one TBK1 subunit capable of efficiently phosphorylating Ser172 of the other TBK1 molecule in the system but not the other subunit within the same dimer, as their kinase domains face different directions. However, this model has difficulties explaining the initial step of TBK1 activation since all TBK1 molecules are assumed to be in an inactive conformation initially. A more plausible explanation is that a potential upstream kinase(s) promotes Ser172 phosphorylation in the initial step of TBK1 activation. Although the most direct mechanism of TBK1 activation in cancer cells remains not fully understood, several signaling pathways have been reported to induce TBK1 activation. In this minireview, we summarize various mechanisms contributing to TBK1 activation in different cancer cells, with a special focus on direct upstream kinases that promote TBK1 activity by phosphorylating the Ser172 residue.

## 2 TBK1 activation by upstream signaling complexes

**Fig. 1:**
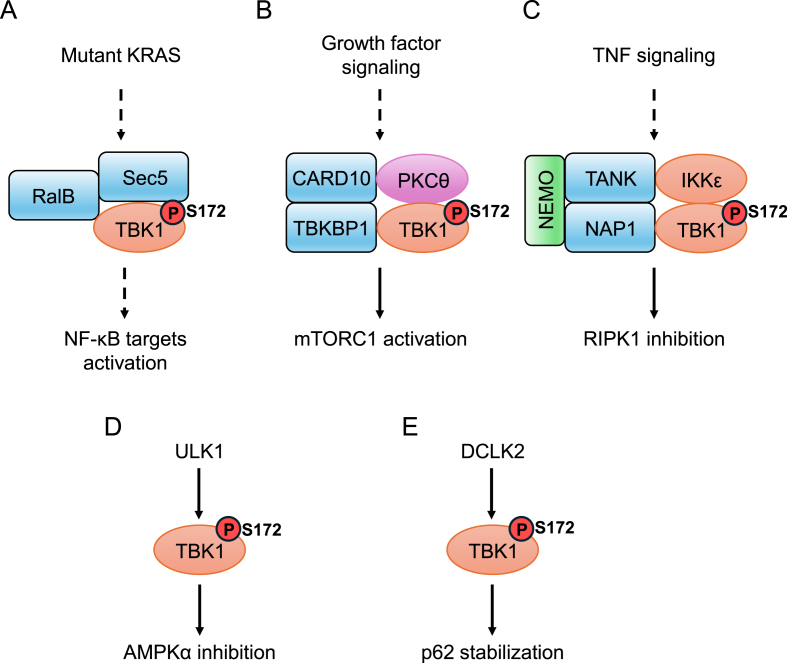
**Activation of TBK1 by different upstream signaling.** A, mutant KRAS can induce TBK1 activation mediated by RalB/Sec5/TBK1 complex, where TBK1 activation may be facilitated by Sec5 taking an open conformation. B, growth factor treatment can promote TBK1 activation through a signaling complex containing TBK1, TBKBP1, CARD10 and PKCθ. TBK1 activation can be promoted by PKCθ mediated phosphorylation of Ser716. C, TNF signaling can induce TBK1/IKKε activation at the TNFR1-SC, which is mediated by adaptor proteins including NEMO, TANK and NAP1. D, in adipocytes, ULK1 can activate TBK1 by directly phosphorylating TBK1 Ser172 site. E, in ccRCC cells, DCLK2 can activate TBK1 by directly phosphorylating TBK1 Ser172 site. Abbreviation: TNF, tumor necrosis factor. TNFR1-SC, TNF receptor 1 signaling complex. ccRCC, clear cell renal cell carcinoma.

RalA and RalB are monomeric Ral GTPases whose activation supports the initiation and maintenance of tumorigenic transformation downstream of Ras signaling (Gentry et al., 2014). TBK1 can be activated in a RalB GTPase-dependent manner (Chien et al., 2006). Sec5, an effector protein of RalB, mediates Ral regulation of dynamic secretory vesicles targeting and tethering. Activation of RalB drives the formation of TBK1/Sec5 protein complex, correlating with TBK1 activation, as measured by *in vitro* phosphorylation of recombinant IRF3. This line of research found that TBK1 could directly phosphorylate Sec5, proposing that Sec5 phosphorylation may stabilize it in an open conformation and facilitate TBK1 activation (Chien et al., 2006) (Fig. 1A). In addition to supporting cancer cell survival, the RalB/Sec5/TBK1 cascade is also required for IRF3 nuclear translocation and Sendai virus-induced IFN-β promoter activation, suggesting that RalB/Sec5 effector complex, as an oncogenesis driver, could also be part of innate immune signaling.

Furthermore, TBK1 activity is selectively essential for the survival of cancer cells with mutant KRAS, identified in a systematic synthetic lethal RNAi screening in NCI-H23 cells, a lung cancer cell line expressing mutant KRAS (Barbie et al., 2009). Suppression of RalB results in a similar synthetic lethality phenotype, suggesting that RalB/TBK1 signaling is crucial in KRAS-dependent cells. This study found that NF-κB anti-apoptotic signaling is selectively activated in KRAS-dependent cells and suppression of TBK1 significantly downregulated multiple anti-apoptotic proteins known as NF-κB targets, including p105, c-Rel and BCL-xL, providing mechanistic insights into this synthetic lethal interaction (Barbie et al., 2009) (Fig. 1A).

Ras-RalB-mediated TBK1 activation extends to pancreatic cancer. The non-receptor tyrosine kinase Axl was reported to be a TBK1 activator and regulate TBK1-driven epithelial cell plasticity in pancreatic cancer (Cruz et al., 2019; Ludwig et al., 2018). Axl promotes TBK1 activation in a Ras-RalB activity-dependent manner, as activating Axl can increase GTP-bond Ras and RalB (Cruz et al., 2019). One consequence of TBK1 activation downstream of oncogenic Ras signaling is increased autophagy activity. However, autophagy suppresses TBK1 signaling through degradation of the active form of TBK1 that is phosphorylated at Ser172 (Yang et al., 2016). This feedback loop was considered to prevent excessive activation of autophagy induced by TBK1 and limit the production of proinflammatory cytokines and the recruitment of neutrophils and T cells.

TBK1 is also a central mediator of growth factor signaling in lung cancer tumorigenesis. Zhu et al. found that TBK1 can be activated by epidermal growth factor (EGF), insulin or fetal bovine serum (FBS)-induced signaling (Zhu et al., 2019). Growth factors induced TBK1 activation involves the assembly of a signaling complex containing TBK1, TBKBP1, CARD10 and protein kinase C θ (PKCθ). In this complex, PKCθ directly phosphorylates Ser716 on TBK1 C-terminus and stimulates TBK1 autophosphorylation on Ser172, which is required for growth factors-induced mTORC1 activation (Bodur et al., 2018) (Fig. 1B). TBK1 with S716A mutation failed to rescue the growth defect of TBK1-knockdown A549 cells both *in vitro* and *in vivo*, suggesting that phosphorylation of Ser716 is critical for the oncogenic function of TBK1 in lung cancer progression (Zhu et al., 2019). Notably, this study suggested that the roles of TBK1 in tumorigenesis and innate immunity are distinguished by different mechanisms, as growth factors do not stimulate expression of interferon and the TBK1/TBKBP1/CARD10 protein complex is not required for TBK1 activation by pattern recognition receptors or DNA/RNA virus infection.

TBK1, and its homologue IKKε, can also be activated by tumor necrosis factor (TNF) stimulation, which is essential to prevent TNF-induced, RIPK1-dependent cell death (Lafont et al., 2018; Xu et al., 2018). After TNF treatment, TBK1 is activated in a NEMO (also known as IKKγ)-dependent manner. NEMO enables the recruitment and activation of TBK1 at the TNF receptor 1 signaling complex (TNFR1-SC), mediated by adaptor proteins including TANK and NAP1. At the TNFR1-SC, activation of TBK1 and IKKε is partly dependent on the canonical IKK kinases (IKKα/β) and autophosphorylation. Then, TBK1 and IKKε phosphorylate RIPK1 on multiple sites, restricting RIPK1 activity on promoting complex-II formation and consequent cell death (Lafont et al., 2018) (Fig. 1C). This function is observed in multiple cancer cell lines such as A549, U937 and HeLa and could be an important mechanism by which TBK1 promotes cancer cell survival. Correspondingly, targeting TBK1 can enhance tumor cells responses to immune checkpoint blockade by decreasing the cytotoxicity threshold to effector cytokines including TNF (Sun et al., 2023). This regulation is also implicated in development as homozygous loss of TBK1 in mice is embryonic lethal due to severe liver cell death, and viability can be rescued by TNF knock out or expression of an inactive RIPK1 (Bonnard et al., 2000; Hemmi et al., 2004; Perry et al., 2004; Xu et al., 2018).

## 3 TBK1 activation by direct upstream kinases

Although many TBK1 substrates have been identified to mediate its multiple biological functions, little was known about the direct upstream activator of TBK1 for a long time (Durand et al., 2018). The existence of upstream kinases that can phosphorylate TBK1 Ser172 residue was noted early on. BX795, one of the earliest identified TBK1 inhibitors, can block TBK1-mediated IRF3 phosphorylation. Interestingly, although BX795 inhibits TBK1 activity, the phosphorylation level of Ser172 of endogenous TBK1 is not decreased but substantially increased (Clark et al., 2009). With the inhibitor present, this phosphorylation cannot come from TBK1 autophosphorylation, suggesting that an unknown kinase may be activated to phosphorylate TBK1 on Ser172, forming a feedback loop that tries to keep endogenous TBK1 activity at an appropriate level (Clark et al., 2009). Although the authors proposed that members of mitogen-activated protein kinase kinase kinase (MAP3K) or MAP4K family might contribute to Ser172 phosphorylation, no evidence has been reported so far. Discovering this potential upstream kinase(s) will not only contribute to fully understanding the regulatory mechanism of TBK1 activity, but also help to develop novel therapeutic strategies by intervening in TBK1-related signaling pathways.

Members of the IKK-related kinase family, including IKKα/β/ε and TBK1, display high similarity in their kinase domains (Durand et al., 2018). Since TBK1 can autophosphorylate Ser172, it is not surprising that this site may be phosphorylated by other IKK-related kinases such as IKKα or IKK β (Clark et al., 2011; Lafont et al., 2018). However, it is challenging to identify IKK-unrelated kinases that can directly phosphorylate Ser172 due to a lack of clues, especially in cancer.

Unc-51-like autophagy-activating kinase 1 (ULK1) is a cytoplasmic kinase critical for autophagy initiation (Lin & Hurley, 2016; Zachari & Ganley, 2017). Zhao et al. found that ULK1 directly phosphorylates TBK1 on Ser172, which is promoted by catabolic condition-induced activation of AMP-activated protein kinase (AMPK), a key regulator of energy homeostasis (Zhao et al., 2018). This regulation is observed in adipocytes both *in vitro* and *in vivo*. Interestingly, the ULK1/TBK1 signaling axis can inhibit AMPK activity to preserve energy storage, forming a negative feedback loop which may be important to prevent excessive AMPK activation in adipose tissue (Zhao et al., 2018) (Fig. 1D). Consequently, adipocyte-specific TBK1 knockout attenuates high-fat diet-induced obesity by increasing energy expenditure mediated by AMPK (Zhao et al., 2018). These results suggest a unique role for TBK1 in mediating crosstalk between energy sensing and inflammatory signaling pathways in both over- and undernutrition.

Clear cell renal cell carcinoma (ccRCC) is the most common subtype of human kidney cancer, characterized by the loss of the Von Hippel-Lindau tumor suppressor (VHL) (Hsieh et al., 2017). TBK1 has been reported to be a target for VHL-null ccRCC, with its activity inhibited by the VHL-associated phosphatase PPM1B (Hu et al., 2020). However, the mechanism promoting TBK1 activity in ccRCC remains unclear. Recently an unexpected kinase, doublecortin like kinase 2 (DCLK2), was discovered to be a direct TBK1 activator in ccRCC through a kinome-wide siRNA screening (Hu et al., 2024). DCLK2 is a poorly studied kinase. The very limited reports suggest that DCLK2 is involved in neural migration, survival and cone growth during development and injury (Koizumi et al., 2006; Nawabi et al., 2015). In ccRCC, DCLK2^203^, a specific isoform of *DCLK2* gene, directly phosphorylates TBK1 on Ser172, thereby promoting TBK1 activity (Hu et al., 2024). Overexpression or depletion of DCLK2^203^ can correspondingly promote or inhibit TBK1 activity, as indicated by phosphorylation of Ser172 on TBK1 and Ser366 on p62 (also known as SQSTM1), a downstream target of TBK1 in ccRCC (Hu et al., 2020) (Fig. 1E). Functionally, TBK1 is the major downstream mediator of the oncogenic function of DCLK2 in ccRCC (Hu et al., 2024). Indeed, till now TBK1 is the first clearly identified DCLK2 kinase substrate. Interestingly, the authors also found that STK3 (also known as MST2) can promote TBK1 phosphorylation on Ser172 *in vitro*. However, subsequent experiments suggested that depletion of STK3 did not consistently decrease TBK1 phosphorylation in all ccRCC cells examined (Hu et al., 2024). Therefore, whether and, if yes, in which context, can STK3 regulate TBK1 activity remains elusive.

Our understanding of TBK1 upstream signaling in cancer and other diseases remains incomplete. Additional potential direct TBK1 activators are under investigation, either in tumorigenesis, innate immunity and other TBK1-involved signaling pathways. According to a recent preprint manuscript, double-stranded DNA (dsDNA) and dsRNA can induce TBK1 activation through multiple distinct pathways, in addition to the well-established cGAS/STING or RIG-I/MAVS signaling complex (Ye et al., 2022). For example, even with cGAS or STING depletion, dsDNA treatment still can induce TBK1 Ser172 phosphorylation, albeit at a lower level. Interestingly, cGAS or STING depletion strongly blocks dsDNA-induced, TBK1-mediated IRF3 phosphorylation on Ser386, but does not affect dsDNA-induced, TBK1-mediated p62 phosphorylation on Ser408, suggesting that an unknown upstream activator is involved in dsDNA-induced TBK1 activation, directing TBK1 to p62 signaling independent of the cGAS/STING-TBK1-IRF3 signaling axis (Ye et al., 2022). As an autophagy receptor, p62 plays important roles in autophagy-mediated antiviral immune responses (Choi et al., 2018). Therefore, simultaneous activation of the two downstream pathways by TBK1 may enhance antiviral immunity through cooperative mechanisms.

## 4 Conclusion

Here we reviewed the activation of TBK1 in cancer cells induced by multiple signaling components, such as RalB/Sec5/TBK1 complex, TBK1/TBKBP1/CARD10/PKC complex and TNFR1-SC complex. We also discussed the direct upstream activators that promote TBK1 activity by phosphorylating the critical Ser172 residue (Fig. 1). The functions of TBK1 and related substrates in tumorigenesis are summarized in Table 1. An intriguing question is whether a direct kinase is required to phosphorylate TBK1 Ser172 for all types of upstream signaling-induced TBK1 activation. For example, previously it was considered that DNA or RNA virus infection-induced TBK1 activation did not require an additional kinase (Zhang et al., 2019). However, recent discoveries have added complexity to this question (Ye et al., 2022). A notable observation is that although TBK1 has many substrates, its upstream signaling pathways remain inadequately explored. Establishing connections between specific upstream signals and downstream effects will be a key direction for future investigation. Given the various functions of TBK1 in physiological and pathological conditions, its substrate specificity may fine-turn TBK1 activity in a context-dependent and spatiotemporal-specific manner. The detailed mechanism behind this substrate specificity is not fully understood. One potential explanation is that upstream signaling may cooperate with additional signaling components to facilitate TBK1 on specific substrate recognition. For example, in the innate immune response, activated TBK1 can phosphorylate a conserved serine site on adaptor proteins (including STING, MAVS and TRIF), creating a docking site for IRF3 (Liu et al., 2015). The recruited IRF3 is then phosphorylated by TBK1. However, these adaptors in innate immune signaling do not appear to be involved in oncogenic signaling-induced TBK1 activation, such as that mediated by DCLK2 or growth factors, which do not trigger IRF3 signaling (Hu et al., 2024; Zhu et al., 2019). DCLK2 has been reported to promote TBK1-p62 signaling axis in ccRCC. Further investigation is needed to determine whether this regulation is facilitated by other components.

**Table 1:** Functions of TBK1 and related substrates in tumorigenesis.

Function of TBK1	Related substrate and target site	Reference
Promoting cell survival in KRAS mutant cancer	NF-kB signaling components (may not be direct)	Barbie et al. (2009)
Promoting growth factors-induced mTORC1 activation in lung cancer	mTOR (Ser2159)	(Bodur et al., 2018; Zhu et al., 2019)
Preventing TNF-induced, RIPK1-dependent cell death in multiple cancers	RIPK1 (multiple sites)	(Lafont et al., 2018; Xu et al., 2018)
Promoting cell proliferation and survival in kidney cancer	p62 (Ser366)	Hu et al. (2020)

It is still far from fully understanding the upstream signaling that induces TBK1 activation, particularly the direct upstream kinase that can activate TBK1. Since TBK1 can be activated at multiple signaling platforms, it is reasonable to expect the discovery of more direct TBK1 upstream kinases in the future. Answers for this question will also help in developing strategies to selectively target TBK1 activity for specific physiological or pathological conditions.

## CRediT authorship contribution statement

**Lianxin Hu:** Writing – review & editing, Writing – original draft, Conceptualization. **Qing Zhang:** Writing – review & editing, Supervision.

## Declaration of competing interest

The authors declare no conflict of interest.
